# An Electric Hot-Water Bottle

**Published:** 1901-11-30

**Authors:** 


### AN ELECTRIC HOT-WATER BOTTLE.

To those who practise in civilised regions where electric lighting is in operation, a suggestion, which we find in the *Philadelphia Medical Journal*, may prove useful—namely, that where the application of heat is desired this may be arranged by the simple plan of wrapping an ordinary electric light bulb, attached to a long flexible conducting cord, in a layer or two of cloth, and applying it to the part. The heat given off is moderate and continuous. For local pains in the chest or the abdomen, and for neuralgic pains in the head, such an arrangement has all the advantages of the hot-water bottle, in addition to which it has the great one of not going cold. It may also be used to "iron" the painful parts in cases of muscular rheumatism or in sciatica. It may be well to remember that the cloth in which the bulb is wrapped should be of woollen fabric or of silk, for the temperature attained by a lamp which is well wrapped up, approaches so nearly to that which will set fire to cotton fabrics that there might be danger if this precaution were neglected.

